# On Variations in the Level of PER in Glial Clocks of *Drosophila* Optic Lobe and Its Negative Regulation by PDF Signaling

**DOI:** 10.3389/fphys.2018.00230

**Published:** 2018-03-19

**Authors:** Jolanta Górska-Andrzejak, Elżbieta M. Chwastek, Lucyna Walkowicz, Kacper Witek

**Affiliations:** Department of Cell Biology and Imaging, Institute of Zoology and Biomedical Research, Jagiellonian University, Krakow, Poland

**Keywords:** circadian clocks, glial oscillators, neuronal pacemakers, PER, PDF, drosophila glia, *Drosophila*

## Abstract

We show that the level of the core protein of the circadian clock Period (PER) expressed by glial peripheral oscillators depends on their location in the *Drosophila* optic lobe. It appears to be controlled by the ventral lateral neurons (LNvs) that release the circadian neurotransmitter Pigment Dispersing Factor (PDF). We demonstrate that glial cells of the distal medulla neuropil (dMnGl) that lie in the vicinity of the PDF-releasing terminals of the LNvs possess receptors for PDF (PDFRs) and express PER at significantly higher level than other types of glia. Surprisingly, the amplitude of PER molecular oscillations in dMnGl is increased twofold in PDF-free environment, that is in *Pdf*^0^ mutants. The *Pdf*^0^ mutants also reveal an increased level of glia-specific protein REPO in dMnGl. The photoreceptors of the compound eye (R-cells) of the PDF-null flies, on the other hand, exhibit de-synchrony of PER molecular oscillations, which manifests itself as increased variability of PER-specific immunofluorescence among the R-cells. Moreover, the daily pattern of expression of the presynaptic protein Bruchpilot (BRP) in the lamina terminals of the R-cells is changed in *Pdf*^0^ mutant. Considering that PDFRs are also expressed by the marginal glia of the lamina that surround the R-cell terminals, the LNv pacemakers appear to be the likely modulators of molecular cycling in the peripheral clocks of both the glial cells and the photoreceptors of the compound eye. Consequently, some form of PDF-based coupling of the glial clocks and the photoreceptors of the eye with the central LNv pacemakers must be operational.

## Introduction

The circadian clocks, displaying molecular oscillations of canonical clock molecules Period (PER) and Timeless (TIM) with a period of ~24 h, are endogenous pacemakers that lay cellular foundation for biological timekeeping (Tataroglu and Emery, 2015). There are two main types of cells that express genes encoding PER and TIM (*per, tim*) in the brain of *Drosophila melanogaster*: the so called clock neurons and the glial cells (Siwicki et al., 1988; Zerr et al., 1990). The former constitute the central pacemaker whereas the latter play the function of peripheral oscillators, similar to photoreceptors of the compound eye and to many cells in non-neural tissues of the head and body (Hardin, 2011; Xu et al., 2011).

In *Drosophila* brain, there are about 150 clock neurons of the central pacemaker distributed in lateral and dorsal clusters that minister different circadian functions (Helfrich-Förster, 2005; Nitabach and Taghert, 2008; Hermann-Luibl and Helfrich-Förster, 2015). There are seven groups in each brain hemisphere, but the most important role in the circadian network and behavioral rhythmicity of flies plays the ventrolateral cluster of clock neurons (LNvs), which includes neurons with small and large cell bodies (Allada and Chung, 2010). The small-lateral neurons (s-LNvs) are crucial for maintaining the circadian activity rhythm (Blanchardon et al., 2001). They project toward the dorsal protocerebrum, where they form short arborizations (Helfrich-Förster, 1998) displaying prominent circadian changes of morphology (Fernández et al., 2008; Gorostiza et al., 2014). The large-lateral neurons (l-LNvs), on the other hand, are not necessary for sustaining the rhythm of activity in constant darkness (DD), but they are essential for light-mediated modulation of arousal and sleep (Sheeba et al., 2008, 2010). It is presumed that their input may be particularly robust, since they display the molecular rhythms of PER and TIM that are phase-advanced and of higher amplitude compared to other clock neurons (Rosato and Kyriacou, 2008). They send projections to the optic lobe and densely arborize on the surface of the second optic neuropil, the medulla (Helfrich-Förster, 1998; Helfrich-Förster et al., 2007). They are, therefore, anatomically well-situated to receiving the light input from the retina of the compound eye (in addition to the one they receive via activation of their photopigment–Cryptochrome; Yoshii et al., 2008) and conveying circadian signals to the optic lobe. They control the output by the paracrine release of the main circadian transmitter - the neuropeptide Pigment Dispersing Factor (PDF) (Helfrich-Förster, 1997; Park et al., 2000), and by signaling via its receptor—PDFR (Renn et al., 1999; Lin et al., 2004; Lear et al., 2005; Shafer et al., 2008; Im and Taghert, 2010; Im et al., 2011). It synchronizes different clusters of clock neurons and the whole circadian network (Lin et al., 2004; Lear et al., 2005; Nitabach et al., 2006; Shafer et al., 2008; Yoshii et al., 2009).

The glial cells, even though much less studied than the clock neurons, have already proved to be integral components of the circadian network (Zwarts et al., 2015). In *Drosophila* brain, like in vertebrates, we discern many different types of glial cells (Edwards and Meinertzhagen, 2010) based on their morphology (Carlson and Saint Marie, 1990; Cantera and Trujillo-Cenoz, 1996), gene expression, and lineage analysis (Ito et al., 1995; Giangrande, 1996; Klämbt et al., 1996; Edwards et al., 2012). The early studies on clock genes expression in *Drosophila* revealed that numerous glial cells display cyclic expression of *per* (Siwicki et al., 1988; Zerr et al., 1990) and *tim* (Peschel et al., 2006), and that the expression of *per* in glia might be sufficient to drive a weak behavioral rhythm (Ewer et al., 1992). Exciting recent works have shown that rhythmic expression of both clock proteins and glia-specific proteins, such as Ebony are involved in regulation of behavioral rhythms (Suh and Jackson, 2007; Ng et al., 2011; Ng and Jackson, 2015). Glial cells of the visual system of Diptera, on the other hand, have been shown to contribute to the circadian plasticity of flies visual system. Epithelial glial cells of the first visual neuropil or lamina display robust rhythmic changes in their volume (Pyza and Górska-Andrzejak, 2004) and in the level of expression of the catalytic subunit of sodium pump, the Na^+^/K^+^-ATPase α subunit (Górska-Andrzejak et al., 2009). Their modulatory input affects both the rhythm of expression of a presynaptic protein Bruchpilot in the lamina synaptic neuropil (Górska-Andrzejak et al., 2013) and the pattern of rhythmic morphological changes of L1 and L2 interneurons, which are the main postsynaptic partners of the compound eye photoreceptors (Pyza and Górska-Andrzejak, 2004; Górska-Andrzejak, 2013).

So far, the glial clocks are known to act downstream of the clock neurons (Suh and Jackson, 2007), but they signal back to them as well (Ng et al., 2011). The circadian rhythmicity, including the rhythmicity of behavior, appears therefore to depend on the glia-neuronal communication and reciprocal interactions (Zwarts et al., 2015; Ng et al., 2016). Nevertheless, the exact nature of mutual influence of the neuronal and glial clocks is far from being fully understood. It is still under investigation how much influence the neuronal pacemakers have on the peripheral glial oscillators and what are the exact neuronal and glial signals that are used in their communication (Zwarts et al., 2015).

Our results reveal heterogeneity of the optic lobe glial clocks. We show that the glial cells situated in the vicinity of the terminals of the circadian clock ventral LNvs may be the most robust molecular oscillators among the glia. We also demonstrate that the clock neurons of the ventrolateral cluster influence the level of PER (the amplitude of the clock) in the glia and in the eye photoreceptors by PDF signaling. Consequently, we propose a novel role for PDF as a potential link between the central and the peripheral clocks in glial and photoreceptor cells. Our study on *Pdf*^0^ mutants suggests that the LNv neurons negatively influence the level of PER in the former and enhance the synchronization among the latter.

## Materials and methods

### Animals

We used the following strains of *D. melanogaster*: wild-type Canton-S (CS), *w*+; *Pdf*^0^ mutant (referred to as *Pdf*^0^; a kind gift from Charlotte Förster, University of Würzburg), *period* mutant (*per*^0^) and *tim*-Gal4 transgenic strain (a kind gift from François Rouyer, Paris Saclay Institute of Neuroscience), as well as other transgenic strains from Bloomington Drosophila Stock Centre (BDSC): *repo*-Gal4 (BDSC, stock no. 7415), *pdfR*-Gal4 (BDSC, stock no. 33070), UAS-pdfR^RNAi^ (BDSC, stock no. 42508), UAS-VAL10- GFP (BDSC, stock no. 35786), UAS-S65T-GFP (BDSC, stock no. 1521), and UAS-mCD8-GFP (BDSC, stock no. 5137). The stocks were maintained on a standard yeast-cornmeal-agar medium, at 25 ± 1°C, under light/dark or day/night conditions (12 h of light and 12 h of darkness, LD 12:12; ZT0 and ZT12 denote the beginning of the day and the night, respectively, ZT—Zeitgeber Time). 7- to 10- days old males were used for each experiment. Following their eclosion, they were divided into two groups which were kept either in LD 12:12, or in DL 12:12 (reversed cycle) for 1 week prior to their decapitation at several time points during the day and night of the 24-h cycle. Flies kept in LD 12:12 were decapitated during the light phase (day) of the cycle, at ZT24/0, ZT1, and ZT4, whereas flies kept in DL 12:12 were decapitated during the dark phase (night) of the cycle, at ZT13, ZT16, ZT19, and ZT21. Flies used for experiments in constant darkness conditions (DD) were entrained in LD 12:12 for 4 days and then transferred to DD for 2 days. On the third day of DD they were decapitated at CT1, CT4, CT13, CT16, CT19, CT21, and CT24 (CT0 and CT12 denote the beginning of the subjective day and the subjective nigh, respectively, CT-Circadian Time).

### Immunolabeling

Experimental flies were immobilized with CO_2_ and decapitated directly in a drop of freshly prepared fixative: the solution of 4% paraformaldehyde (PFA) in 0.1 M Phosphate Buffer (PB). Approximately 30 flies were sacrificed for each data point. After fixation and cryoprotecting infiltration in the solution of 25% sucrose in 0.01 M sodium Phosphate Buffer Saline (PBS), their heads were cut either in the frontal or horizontal plane into 20 μm thick cryosections. Following this, they were incubated with a rabbit polyclonal anti-PER serum (a gift from Ralf Stanewsky, University of Munster; Stanewsky et al., 1997) and a goat anti-rabbit Cy3-conjugated secondary antibody (Jackson ImmunoResearch Laboratories). To visualize the clock neurons that synthetize the neuropeptide PDF, we used a polyclonal rabbit anti-β-PDH serum, which recognizes the insect PDF (a gift from Ezio Rosato, University of Leicester; Dircksen et al., 1987). We also used anti-REPO (8D12) and anti-BRP (nc82) mouse antibodies (Developmental Studies Hybridoma Bank, DSHB). The fluorescence staining was performed with Cy3-conjugated secondary antibodies. Fluorescently labeled tissue was examined using Zeiss LSM 510 Meta confocal microscope following extensive washing and mounting in a Vectashield or DAPI-containing Vectashield (Vector).

### Quantification of immunolabeling

*Drosophila melanogaster* heads that were collected at various time points (of the same 24-h cycle) were fixed, processed and immunolabeled in parallel, under the same conditions. Then, their images were acquired at non saturated settings, using identical image acquisition parameters for all data points (time points). The circadian changes in the intensity of PER-specific immunolabeling corresponding to the changes in the amount of PER protein, were measured in the glial cells of the optic lobe, as well as in the lateral clock neurons and in the photoreceptors of the compound eye. The level (brightness) of fluorescence represented by the Mean Gray Value (the sum of the gray values of all pixels in the selected area, divided by the number of pixels within the selection) was measured using ImageJ software (NIH, Bethesda). In this software, the range of gray values (between the *Min* and *Max*) in 8-bit images is divided into 256 bins. The background signal was subtracted.

### Statistics

To allow the comparisons between data from different experiments, which revealed certain differences in the intensity of labeling, the data were presented as a percentage of the highest value (100%) that was obtained in a given experiment. All the data were statistically analyzed using the Shapiro–Wilk *W*-test for normality. The differences between experimental groups (ZT/CT time points) were calculated based on the mean of measurements obtained from 7 to 12 individuals within a group (decapitated at the particular time point). The statistical significance of differences between groups was estimated using either ANOVA, or the nonparametric counterpart of ANOVA—Kruskal–Wallis test (one-way test) followed by *post-hoc* Multiple Comparison Test. In each analysis a probability value of *p* < 0.05 was set for significant differences.

## Results

**Figure 1:**
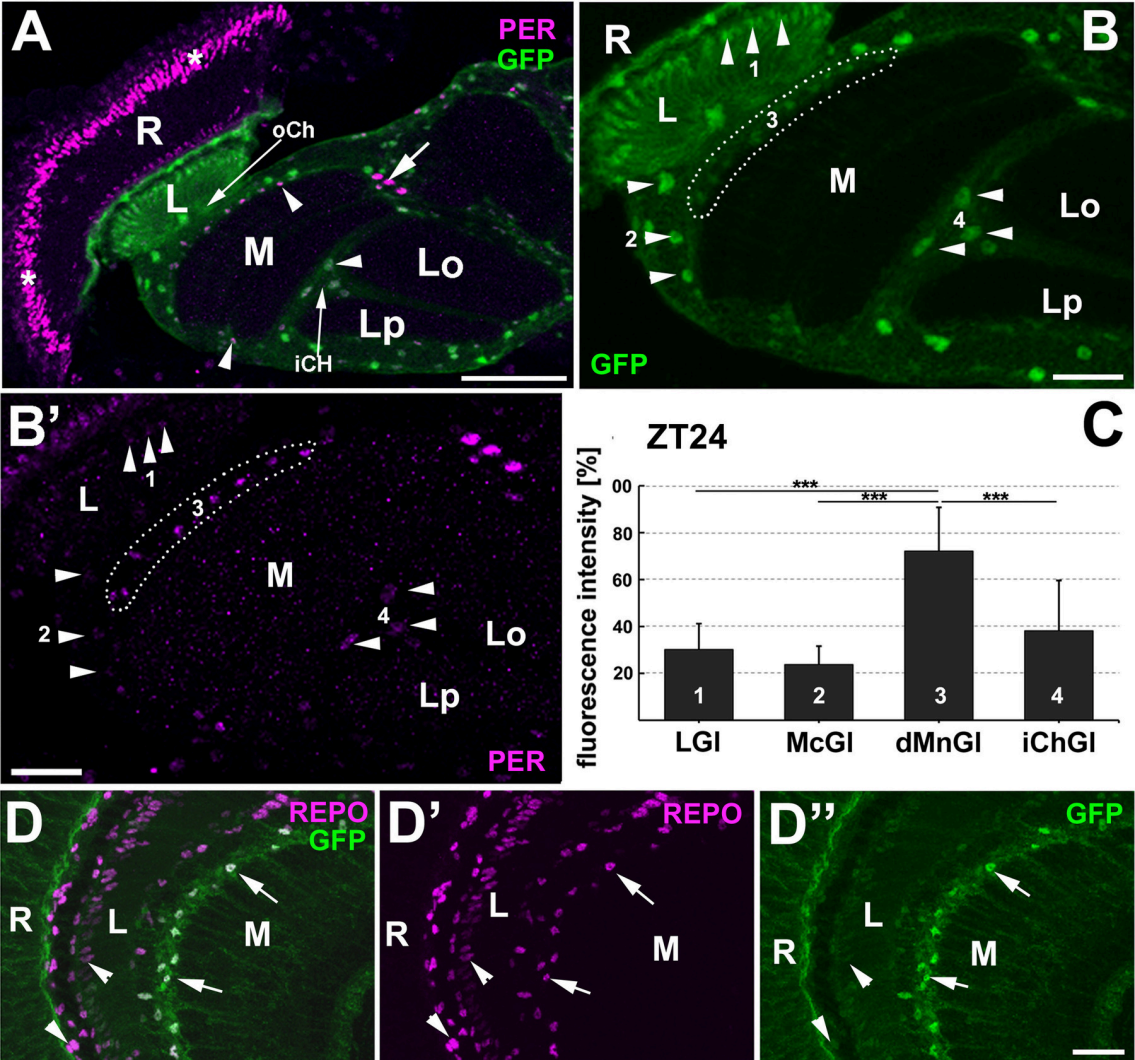
**(A)** Compound eye retina and underlying optic lobe of flies with targeted expression of Green Fluorescent Protein (GFP) to glial cells (*repo*-Gal4/UAS-S65T-GFP) and immunolabeled with anti-PER serum. PER-positive nuclei (magenta) belong to the clock cells of the lateral protocerebrum, the so called ventral lateral neurons-LNvs (arrow), the compound eye photoreceptors (asterisks) and the glial cells (arrowheads). R-retina, L-lamina, M-medulla, Lo, and Lp- two parts of the lobula complex, oCh- outer chiasm and iCh-inner chiasm. Scale bar: 50 μm. **(B)** The glial cells selected for measurements of PER-specific immunofluorescence (arrowheads): the epithelial glial cells of the lamina neuropil (LGl, 1), the medula cortex glia (McGl, 2), the distal medulla glia (dMnGl, 3), and the inner chiasm glia (iChGl, 4). R-retina, L-lamina, M-medulla, Lo, and Lp- two parts of the lobula complex. Scale bar: 20 μm. **(B')** PER-specific immunofluorescence of cells shown in **(B)**. Scale bar: 20 μm. **(C)** The average level of PER-specific immunofluorescence (±*SD*) in the nuclei of LGl (1), McGl (2), dMnGl (3), and iChGl (4) in the optic lobe of CS flies at the end of the night (ZT24). The statistically significant differences are marked by asterisks (^***^p ≤ 0.001). **(D–D”)** The lamina and medulla of flies with targeted expression of GFP (green) to *tim* expressing cells (*tim*-Gal4/UAS-S65T-GFP) and immunolabeled using 8D12 Mab against pan-glial REPO marker (magenta). Among many glial cells that are marked by REPO-specific immunofluorescence, only dMnGl (arrows) express the GFP reporter on such a high level. The low level of GFP is seen in the nuclei of other glial cells, especially glia of the lamina cortex, or lamina neuropil (arrowheads). R, retina; L, lamina; M, medulla. Scale bar: 20 μm.

PER-specific immunolabeling was observed in the usual locations (Siwicki et al., 1988; Zerr et al., 1990; Ewer et al., 1992) of CS brains, as well as in brains of *repo*-Gal4/UAS-S65T-GFP transgenic flies (Figure 1) that were sectioned at the end of the dark phase/night (ZT24). It was detected in the nuclei of circadian pacemaker neurons (of lateral and dorsal groups; LNs and DNs), compound eye photoreceptors, and numerous glial cells (Figure 1A). Such a pattern of labeling in CS and *repo*-Gal4/UAS-S65T-GFP transgenic flies (Figure 1) confirmed the specificity of the applied serum, which was further supported by lack of labeling in *per*^0^ mutant—a negative genetic control.

### dMnGl display the highest level of PER of all the glia

The nuclei of PER-immunoreactive glial cells were found in the whole brain of *D. melanogaster* (Figure 1A). When checked on thin (20 μm) cryosections, the intensity of their immunolabeling (reflecting the amount of nuclear PER) was discovered to vary significantly, depending on the location of glia in the brain, in other words—on the type of glia. The largest differences were observed between glia of the first (lamina) and the second (medulla) visual neuropils (Figure 1). PER-specific immunofluorescence was bright in glia of the medulla, while it was barely detectable in glial cells of the lamina (LGl). Interestingly, the highest level of immunofluorescence was observed in the small nuclei of glia inhabiting the distal part of the medulla (hereafter referred to as distal medulla neuropil glia, dMnGl), whose cell bodies are located precisely on the border between the cortex and the neuropil of the medulla (Figures 1A–C). The average level of PER-specific immunofluorescence in the nuclei of dMnGl was 58% higher than in the lamina neuropil glia (LGl), 67% higher than in the cortex/satellite glia of medulla (McGl), and 47% higher than in the tract glia of the inner chiasm (iChGl) (Kruskal–Wallis Test: H[3, *N* = 59] = 32.5, *p* = 0.00001, followed by Multiple Comparison Test, *p* = 0.0008, *p* = 0.000003, and *p* = 0.0004, respectively; Figure 1). These cells also expressed the highest level of GFP reporter of *tim*, when examined in *tim*-Gal4/UAS-S65T-GFP transgenic flies (Figures 1D–D”). The differences in the expression level of GFP reporter of *tim* (driven by *tim*-Gal4 pan-circadian driver) and the intensity of PER-specific staining in different types of glia indicate that the population of glial clocks is heterogeneous. The particularly high levels (as far as the glial cells are concerned) of PER and TIM in dMnGl imply high-amplitude cycling of the clock proteins.

### dMnGl are weaker oscillators than neuronal clocks, but work in phase with them

**Figure 2:**
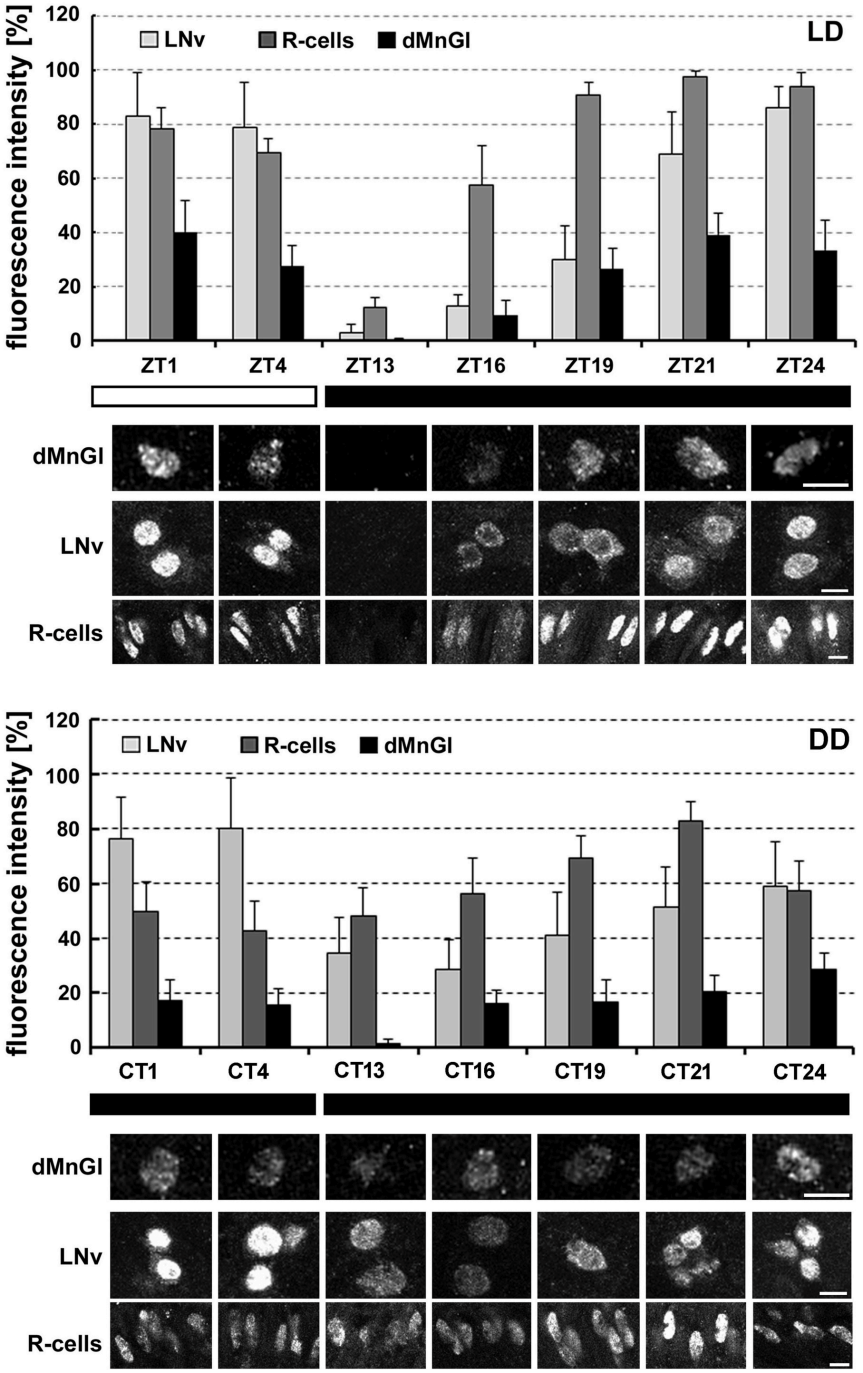
The daily (LD) and circadian (DD) rhythms in PER-specific immunofluorescence (average ± *SD*) as the measure of PER molecular oscillations in the nuclei of dMnGl, the large-lateral ventral pacemaker neurons (LNv) and photoreceptors of the compound eye (R-cells) in the optic lobe of CS flies. Like in the LNv neurons and the R-cells, the average level of PER-specific fluorescence in dMnGl changed significantly in the course of the day in LD (dMnGl: Kruskal–Wallis Test: H [6, *N* = 67] = 48.7, *p* = 0.00001; LNvs: Kruskal–Wallis Test: H [6, *N* = 73] = 58.8, *p* = 0.00001; R-cells: Kruskal–Wallis Test: H [6, *N* = 70] = 59.8, *p* = 0.00001), and in DD (dMnGl: Kruskal–Wallis Test: H [6, *N* = 69] = 36.8, *p* = 0.00001; LNvs: Kruskal–Wallis Test: H [6, *N* = 110] = 65.8, *p* = 0.00001; R-cells: Kruskal–Wallis Test: H [6, *N* = 70] = 41.2, *p* = 0.00001), but with considerably smaller amplitude. Below the charts: exemplary images collected at different time points (ZTs or CTs), showing the peak and trough accumulation of PER in the nuclei of dMnGl, LNvs, and R-cells. Scale bar: 5 μm. White and black bars on the bottom indicate light and dark periods, respectively.

Daily changes of nuclear PER-specific immunofluorescence (reflecting PER rhythmic accumulation in the nucleus) were generally the same in the nuclei of dMnGl as in the nuclei of clock cells of the lateral protocerebrum (LNvs), or in the photoreceptors of the eye (R-cells; Figure 2). The fluorescence was the most intense at the end of the night and at the beginning of the day (ZT24, ZT1), and undetectable (the same as in the surrounding cytoplasm) at the beginning of the night (ZT13; Figure 2), which confirmed the similarity of PER nuclear accumulation patterns in neurons and glial cells. dMnGl were in phase with rhythms of the LNvs and the R-cells, suggesting that they are coupled with the circadian timing system. Even though the oscillatory pattern of the glia was similar to that of the neuronal oscillators (cf. Figure 2), the average daily level of nuclear immunofluorescence in dMnGl was 52% lower than in the LNvs and 65% lower than in the R-cells (Kruskal–Wallis Test: H [2, *N* = 210] = 59.7, *p* = 0.00001, followed by Multiple Comparison Test, *p* = 0.00002 and *p* = 0.000001, respectively). The considerable differences in the level of PER-specific immunofluorescence in dMnGl and LNvs or R-cells were observed at each of the time points of LD cycle, at which the nuclear accumulation of PER can normally be observed (ZT16-ZT4; Figure 2).

In DD conditions the amplitude of oscillations of PER expression was smaller in all three types of cells (Figure 2). The maximum levels of fluorescence (at CT1, CT4, CT21, and CT24) were reduced (e.g., in the R-cells and dMnGl at CT1 and CT4), while the minimum levels (e.g., in the R-cells and the LNvs at CT13) were increased with respect to LD (Figure 2). The elevation of fluorescence intensity at CT13, however, was not observed in dMnGl. The average daily level of fluorescence in dMnGl was on average 70% lower than in the LNvs and in the R-cells (Kruskal–Wallis Test: H [2, *N* = 249] = 130.1, *p* = 0.0001, followed by Multiple Comparison Test, *p* = 0.00001 in case of both dMnGl vs. LNvs and dMnGl vs. R-cells). Hence, the main difference between dMnGl and the neuronal oscillators (LNvs and R-cells) concerns mainly the amplitude of PER oscillations.

### dMnGl possess receptors for PDF (PDFRs)

**Figure 3:**
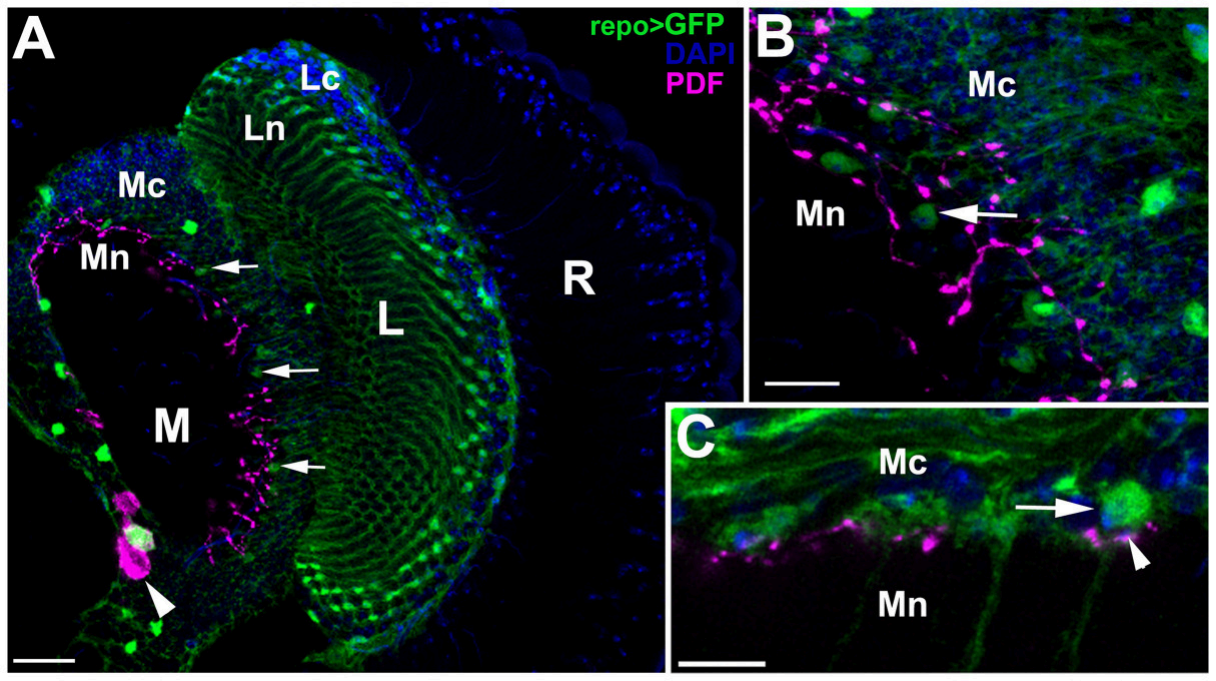
Confocal images of the optic lobe of *repo*-Gal4/UAS-S65T-GFP transgenic flies showing cytoplasmic and nuclear expression of GFP (green) in glial cells, in combination with anti-PDF immunolabeling (magenta), and DAPI nuclear labeling (blue). **(A)** The location of dMnGl in relation to PDF-immunoreactive varicosities of the LNvs on the medulla neuropil surface in frontal section of the optic lobe. PDF-positive cell bodies of the LNvs (arrowhead) are located in the accessory medulla, whereas their optic lobe terminals reside in the distal part of the medulla neuropil, where small nuclei of dMnGl (arrows) are located. Scale bar: 20 μm. **(B)** Magnification of dense varicose arborization of PDF-positive fibers of the LNvs and the nuclei of dMnGl (arrow). Scale bar: 10 μm. **(C)** The horizontal section reveals that PDF releasing terminals (arrowhead) are located right beneath the medulla cortex, in close proximity to dMnGl cell bodies (arrow). R, retina; L, lamina; M, medulla; Lc, lamina cortex; Ln, lamina neuropil; Mc, medulla cortex; Mn, medulla neuropil. Scale bar: 50 μm.

The distinguishing feature of dMnGl is the location of their cell bodies in the neighborhood of the optic lobe terminals of the LNv neurons, which secrete the neuropeptide PDF (Figure 3). This anatomical proximity enables the direct and strong influence of the LNvs on the circadian function of dMnGl. To find out whether the particularly high level of PER in dMnGl might result from this proximity (Figures 3B,C) and this influence, we checked if (i) dMnGl were equipped with receptors for PDF (PDFRs) and (ii) whether the level of PER was lower in dMnGl of *Pdf*^0^ mutants (due to PDF absence) and of *repo*-Gal4 /UAS-pdfR^RNAi^ flies (due to RNAi-driven silenced expression of PDFRs in glia).

**Figure 4:**
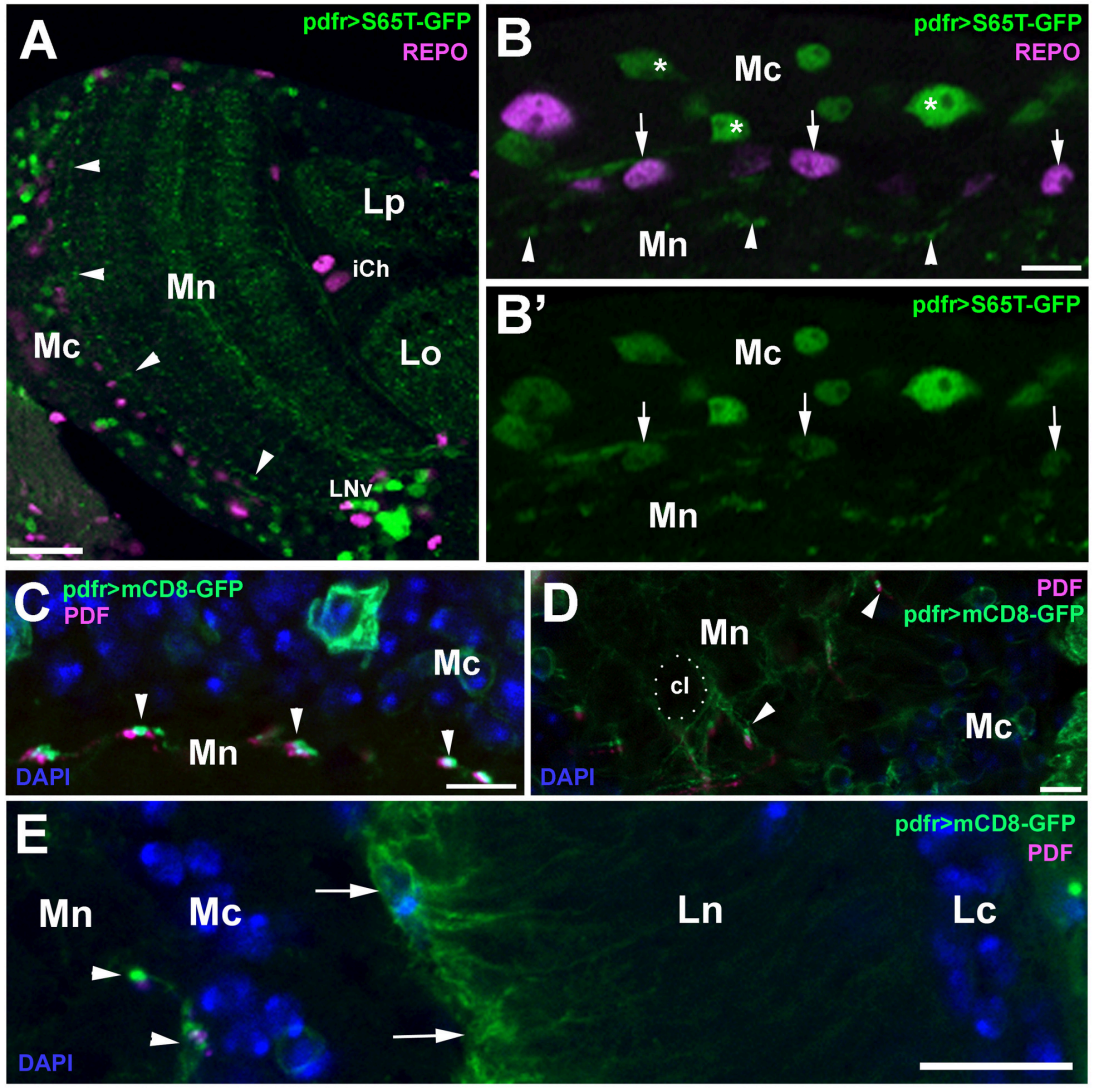
An overview of *pdfR*-Gal4 expression pattern in *Drosophila* optic neuropils. **(A–B')** The medulla of *pdfR*-Gal4/UAS-S65T-GFP flies immunolabeled with anti-REPO Mab (magenta). **(A)** The cytoplasmic and nuclear expression of S65T-GFP reporter (green) shows numerous nuclei and processes of cells that express PDFR, the cell bodies (LNv) and terminals (arrowheads) of the LNvs among them. Lo and Lp-parts of the lobula complex, iCh-inner chiasm. Scale bar: 20 μm. **(B,B')** Higher magnification of the area at the interface of medulla cortex (Mc) and medulla neuropil (Mn) reveals the presence of S65T-GFP reporter **(B')** is REPO-positive nuclei of glia **(B)**, which due to their location must belong to dMnGl (arrows). The terminals of the LNvs are marked by patches of cytoplasmic GFP (arrowheads). GFP-positive, but REPO negative nuclei (asterisks) belong to PDFR-expressing neurons. **(C,D)** The distal medulla of *pdfR*-Gal4/UAS-mCD8-GFP flies immunolabeled using anti-PDF antibodies (magenta). Mc, medulla cortex; Mn, medulla neuropil. **(C)** Membranous expression of mCD8-GFP reporter reveals that the sites (arrowheads) of PDFR expression (green) and PDF release (magenta) are localized next to each other on the LNv terminals. **(D)** PDFR-expressing processes (green) encircle the medulla columns (cl). There are PDF releasing varicosities (magenta) visible on some of these processes (arrowheads). Scale bar for **(B–D)**: 5 μm. **(E)** Expression of mCD8-GFP in the marginal glia (arrows) localized at the margin of the lamina neuropil (Ln) in relation to the LNv terminals (arrowheads) in the distal medulla. Lc, lamina cortex; Mc, medulla cortex; Mn, medulla neuropil. Scale bar: 10 μm.

To resolve the first issue, we investigated in detail the pattern of expression of *pdfR*-Gal4 driver (which reflects the endogenous *pdfR* expression according to Lear et al., 2009), in the region of dMnGl residence. It turned out that the cytoplasmic and nuclear GFP in *pdfR*-Gal4/UAS-S65T-GFP transgenic flies exposed dMnGl (Figures 4A–B'), which indicated the presence of PDFRs in these cells. The level of GFP fluorescence in their nuclei was, however, by 70–78% lower than in the nuclei of other GFP-positive cells localized in their proximity, such as the LNvs and other neurons (Figures 4B,B'). Even though dMnGl express relatively lower amounts of PDFR, they must belong to the LNv target cells, being not only conveniently positioned in the vicinity of the PDF releasing terminals of the LNvs (Figure 3), but also capable of receiving the PDF conveyed circadian information (Figures 4B,B').

The GFP expression controlled by *pdfR*-Gal4 driver exposed also the varicose network of the LNv terminals on the surface of the medulla neuropil (Figure 4A). Their examination in the medulla of *pdfR*-Gal4/UAS-S65T-GFP (cytoplasmic GFP) and *pdfR*-Gal4/mCD8-GFP (membranous GFP) transgenic flies revealed the presence of much brighter spots of green fluorescence—presumably patches of PDFR aggregation (Figures 4A,C). Interestingly, these bright PDFR patches were settled right next to sites of PDF release immunolabeled with anti-PDF Ab (Figure 4C). The LNv terminals, which both release PDF and receive PDF-conveyed information, seem to envelope the center of each unit (column) of the medulla neuropil (Figure 4D). Consequently, the processes that build the medulla column (with dMnGl processes among them) must receive (directly or indirectly) the PDF-conveyed synchronizing signals sent to the optic lobe by the LNv pacemakers. Each medulla column is innervated by PDF neurons and comprises processes that possess PDFRs. It appears, however, that PDF diffuses as far as the proximal part of the first optic neuropil or lamina, since the lamina marginal glia that reside at that part of neuropil strongly express PDFRs (Figure 4E).

### The level of PER increases in glia of *Pdf*^0^ mutants

**Figure 5:**
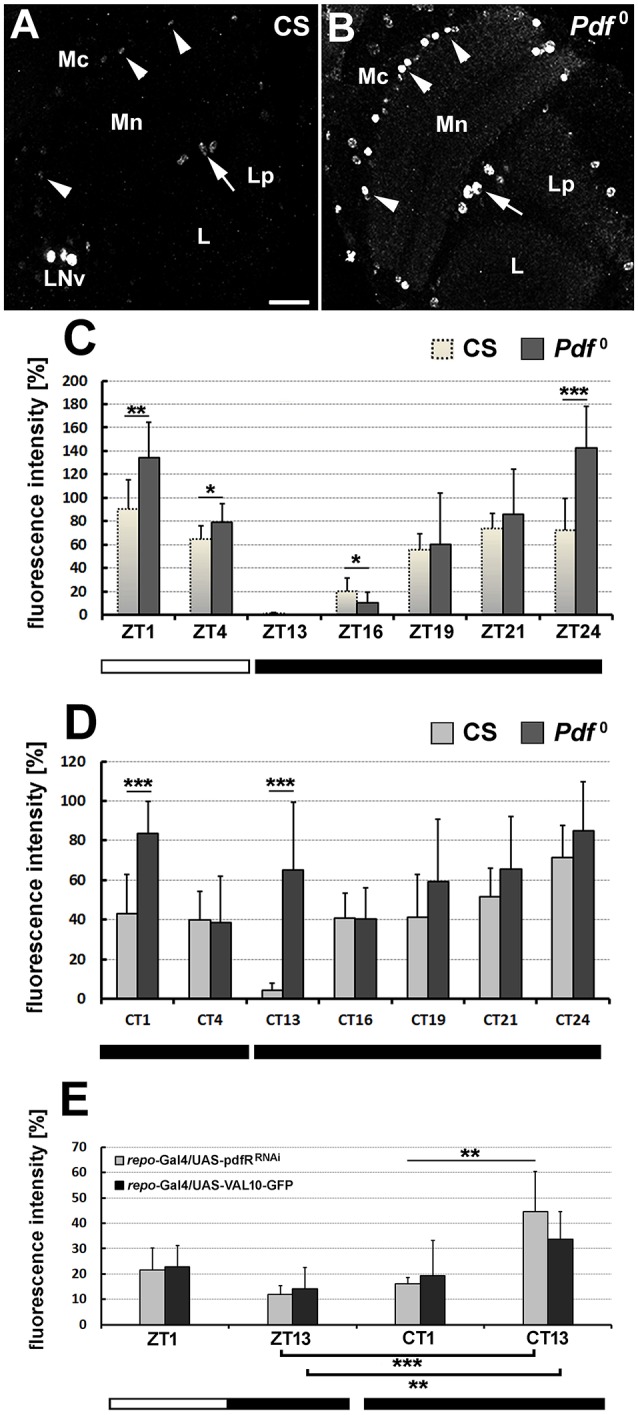
The daily rhythm in the intensity of PER-specific immunofluorescence as the measure of PER molecular oscillations in optic lobes of CS and *Pdf*^0^ flies. **(A,B)** Horizontal sections of optic lobes of flies sacrificed at ZT24. Due to considerable differences in the level of PER immunolabeling in dMnGl (arrowheads) and iChGl (arrows) of CS and *Pdf*^0^, the images collected at the same image acquisition parameters show the nuclei of CS glia on the verge of visibility, when the nuclei of *Pdf*^0^ glia are shown very clearly (with the signal being almost saturated). Mc, medulla cortex; Mn, medulla neuropil; L, lobula; Lp, lobula plate; LNv, ventral lateral neurons. Scale bar: 20 μm. **(C)** Daily profile of anti-PER labeling of *Pdf*^0^ dMnGl (Kruskal–Wallis Test: H [6, *N* = 71] = 55.95, *p* = 0.00001, followed by Multiple Comparison Test: ZT13 vs. ZT1 [*p* = 0.000002], ZT4 [*p* = 0.03], ZT21 [*p* = 0.004], and ZT24 [*p* = 0.00001], also ZT16 vs. ZT1 [*p* = 0.00005], ZT21 [*p* = 0.049], and ZT24 [*p* = 0.00001]) to compare with the profile of CS dMnGl (Kruskal–Wallis Test: H [6, *N* = 66] = 48, *p* = 0.00001 followed by Multiple Comparison Test: ZT13 vs. ZT1 [*p* = 0.000001], ZT4 [*p* = 0.004], ZT21 [*p* = 0.00003], and ZT24 [*p* = 0.001], also ZT16 vs. ZT1 [*p* = 0.0001], ZT21 [*p* = 0.002], and ZT24 [*p* = 0.04], ^*^p ≤ 0.05, ^**^p ≤ 0.01, ^***^p ≤ 0.001). **(D)** The circadian profile of anti-PER labeling of *Pdf*^0^ dMnGl (Kruskal–Wallis Test: H [6, *N* = 74] = 22.62, *p* = 0.0009, followed by Multiple Comparison Test: CT1 vs. CT4 [*p* = 0.02] and CT16 [*p* = 0.02], as well as CT24 vs. CT4 [*p* = 0.003], and CT16 [*p* = 0.005]) to compare with the profile of CS dMnGl (Kruskal–Wallis Test: H [6, *N* = 69] = 36.8, *p* = 0.00001 followed by Multiple Comparison Test: CT13 vs. CT1 [*p* = 0.008], CT4 [*p* = 0.04], CT16 [*p* = 0.03], CT19 [*p* = 0.02], CT21 [*p* = 0003], and CT24 [*p* = 0.000001], ^*^p ≤ 0.05, ^**^p ≤ 0.01, ^***^p ≤ 0.001). **(E)** The average level (±*SD*) of PER-specific immunofluorescence in dMnGl of *repo*-Gal4/UAS-pdfR^RNAi^ at ZT1/ZT13 and CT1/CT13.

In order to account for the influence of PDF on the clock mechanism of dMnGl, we checked whether the level of nuclear PER in dMnGl would be lower in *Pdf*^0^ mutants than in CS flies. Contrary to our expectations, however, in the absence of PDF the level of PER was higher (Figures 5A,B). In LD conditions, PER-specific immunofluorescence in dMnGl of *Pdf*^0^ with respect to CS was 50% higher at ZT24 (*t*-test, *t* = −4.86, df = 18, *p* = 0.0001), 32% higher at ZT1 (*t*-test, *t* = −3.5, df = 18, *p* = 0.003), and 18.7% higher at ZT4 (*t*-test, *t* = −2.2, df = 15, *p* = 0.04). It was 50% lower in *Pdf*^0^ than in CS only in the middle of the night, at ZT16 (*t*-test, *t* = 2.2, df = 19, *p* = 0.04), suggesting a delay in nuclear aggregation of PER in *Pdf*^0^ glia (Figure 5C).

In DD conditions (Figure 5D) PER-specific immunofluorescence in dMnGl of *Pdf*^0^ showed significant increase with respect to CS at CT1 (48%; *t*-test, *t* = −4.6, df = 16, *p* = 0.003) and CT13 (93%; *t*-test, *t* = −5.5, df = 20, *p* = 0.00002). The high level of fluorescence at CT13 was rather unexpected, as in CS it is usually the lowest at this time of the day in both LD and DD (Figure 2). This high level of fluorescence, however, was accompanied by the highest dispersion of results obtained from different individuals. It may imply that the population of *Pdf*^0^ was not well synchronized (which typically shows up at the beginning of the day or the night).

The increase of PER at CT13 (by 73% with respect to ZT13) was also observed in dMnGl of the flies with silenced expression of PDF receptors in glia (*repo*-Gal4/UAS-pdfR^RNAi^, Figure 5E). The level of PER was 64% higher than at CT1 (Kruskal–Wallis Test: H [3, *N* = 36] = 23.1, *p* = 0.0001, followed by Multiple Comparison Test: *p* = 0.00003 for CT13 vs. ZT13 and *p* = 0.004 for CT13 vs. CT1). The increase at CT13 (58%; with respect to ZT13) was also observed in the control flies (*repo*-Gal4/UAS-VAL10-GFP). In this case, however, the fluorescence at CT13 was not significantly higher than at CT1 (Kruskal–Wallis Test: H [3, *N* = 38] = 13.9, *p* = 0.003, followed by Multiple Comparison Test: *p* = 0.002 for CT13 vs. ZT13 and *p* = 0.08 for CT13 vs. CT1; Figure 5E). Also, the lack of statistically significant differences between the experimental and the control flies observed at all the studied time points indicates that the silencing of PER expression in these flies was not strong enough to bring up visible changes of fluorescence.

**Figure 6:**
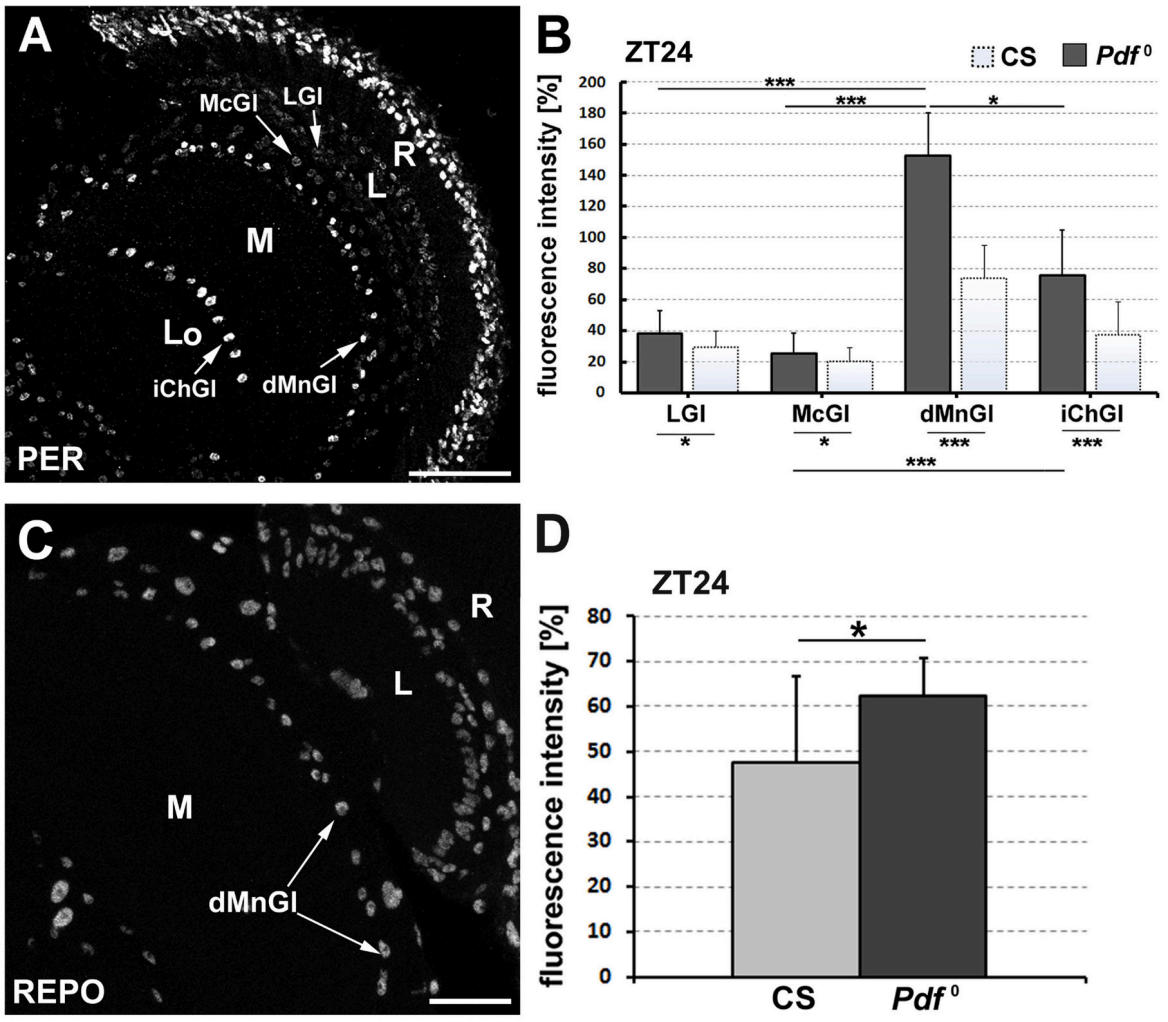
**(A)** The confocal image of *Pdf*^0^ optic lobe displaying type-dependent differences in the level of PER-specific immunofluorescence in the optic lobe glial cells at the end of the night. These differences are similar to those observed in the optic lobe of CS (cf. Figure 1B') but better visible. The strongest signal was observed in dMnGl and iChGl of the medulla neuropil, whereas the weakest was seen in McGl of the medulla cortex and in the lamina. R, retina; L, lamina; M, medulla; Lo, lobula complex. Scale bar: 20 μm. **(B)** The average level of PER-specific immunofluorescence (±*SD*) in the nuclei of dMnGl, LGl, McGl, and iChGl of *Pdf*^0^ flies (dark bars) at ZT24, to compare with the signal in the same types of glia in CS flies (light bars) (^*^p ≤ 0.05, ^***^p ≤ 0.001). **(C)** Horizontal section of the optic lobe of CS flies immunolabeled for REPO. dMnGl-distal medulla neuropil glia, R-retina, L-lamina, M-medulla. Scale bar: 20 μm. **(D)** The average level of REPO (±*SD*) in dMnGl of CS and *Pdf*^0^ at ZT24. Glia od *Pdf*^0^ mutants display higher level of REPO than glia of Canton-S (^*^p ≤ 0.05).

*Pdf*^0^ mutation affected the level of PER also in glia of other locations (Figure 6A). At the end of the night, the level of PER-specific immunofluorescence in iChGl (like in dMnGl) was twice as strong as in their counterparts of CS (*t*-Test, *t* = 6.9, *p* = 0.0000001, and *t* = 3.8, *p* = 0.0008, respectively). In the case of LGl, there was a smaller (23%), but statistically significant (Mann–Whitney Test, *U* = 51, *p* = 0.03) increase in the intensity of fluorescence in *Pdf*^0^ (Figure 6B). This increase considerably improved detectability of LGl in *Pdf*^0^ lamina probed for PER presence. (PER-specific staining in CS lamina was usually faint and less reproducible than in other parts of the brain). Finally, the immunofluorescence in McGl of *Pdf*^0^ was only 20% stronger than in McGl of CS (Mann–Whitney Test, *U* = 58, *p* = 0.3) (Figure 6B). Since PER-specific immunofluorescence increased significantly in *Pdf*^0^ glia of all considered locations in the optic lobe, it appears as though PDF-conveyed signals from the LNv pacemakers attenuated the expression of PER in glia in the wild type flies. Consequently, the CS LNv pacemakers appear to negatively influence the amplitude of molecular oscillations in glial clocks.

The comparison of PER level between different types of glia (dMnGl, LGl, McGl, and iChGl) in *Pdf*^0^ mutants (Figure 6B) not only confirmed the presence of type-related differences (Kruskal–Wallis Test: H [3, *N* = 74] = 53.2, *p* = 0.00001) observed initially in CS optic lobe (Figure 1C), but it showed them more clearly due to general increase of PER-specific immunofluorescence. Like in CS flies, the level of PER was the highest in the case of dMnGl (Figures 6A,B). It was 75, 83, and 50% higher in dMnGl than in LGl (*p* = 0.0000001), McGl (*p* = 0.0000001), and iChGl (*p* = 0.03), respectively (Figure 6B). The differences between dMnGl and LGl or McGl increased by 17 and 16% with respect to the corresponding differences in CS. The difference between the iChGl and McGl in *Pdf*^0^ was also statistically significant (*p* = 0.001). Hence, the differences in PER-labeling (PER level) observed between varied types of CS glia appear to be enhanced in *Pdf*^0^ mutants (Figure 6B).

### The level of repo increases in glia of *Pdf*^0^ mutants

Next, we wanted to find out whether differences in glial PER could affect glia-specific functions. Therefore, we checked if the elevation of glial PER in *Pdf*^0^ mutants was accompanied by alterations in the level of the major glial regulator—the glia-specific homeodomain transcription factor reversed polarity (REPO). The comparative analysis of REPO-specific immunofluorescence (Figure 6C) in the nuclei of dMnGl of CS and *Pdf*^0^ at ZT24 indeed revealed the 24% increase of the signal in dMnGl of *Pdf*^0^. The difference between the levels of REPO in dMnGl of CS and in *Pdf*^0^ (Figure 6D) was not as big as the respective difference in the level of glial PER (50% difference in PER-specific immunofluorescence). It was, however, statistically significant (Mann–Whitney Test, *U* = 26, *p* = 0.04). Therefore it appears that the REPO-controlled glial functions are maintained at higher level in dMnGl of *Pdf*^0^ than in CS flies. Moreover, they are possibly modulated in the circadian manner by neuronal pacemakers (at least by the PDF-releasing LNv neurons).

### R-cells of *Pdf*^0^ display de-synchrony of PER molecular rhythm

**Figure 7:**
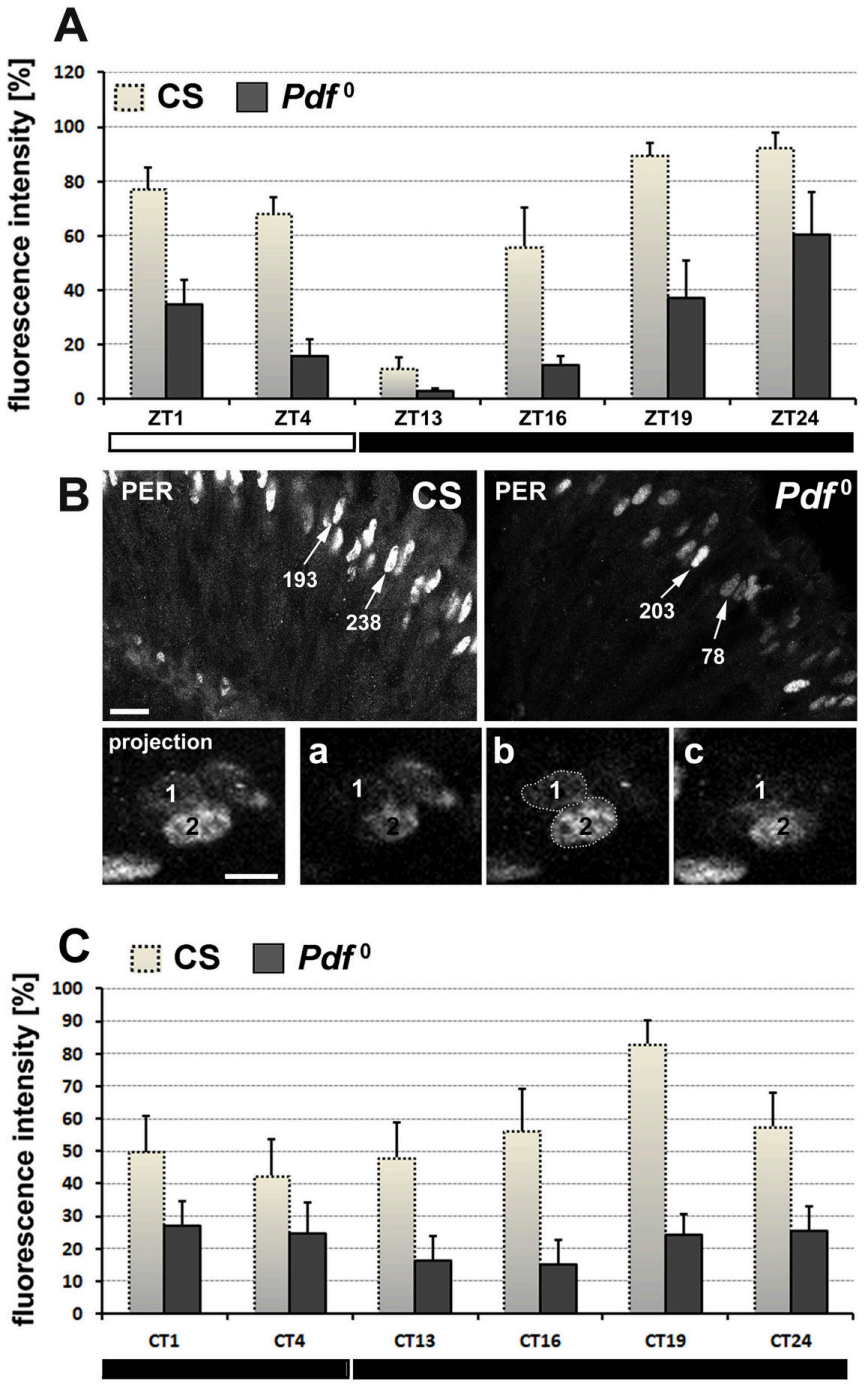
**(A)** The average level (±*SD*) of PER-specific fluorescence in the nuclei of compound eye photoreceptors (R-cells) of CS and *Pdf*^0^ at different ZTs of LD12:12. **(B)** Fragments of CS and *Pdf*^0^ retinas immunolabeled using anti-PER serum. The differences in the level of PER-specific fluorescence among the R-cells of *Pdf*^0^ are much bigger than among the R-cells of CS, which show the annotations of the Mean Gray Value for the nuclei indicated by arrows. Lower panel: the projection of three **(a–c)** consecutive optical sections of the two nuclei of *Pdf*^0^ R-cells, marked as 1 and 2. They display the same level of PER-specific fluorescence (1-low and 2-high) throughout their whole depth—at all three optical sections **(a–c)**. Scale bar: 10 μm. **(C)** The average level (±*SD*) of PER-specific fluorescence in the R-cells nuclei of CS and *Pdf*^0^ at different CTs (CS: Kruskal–Wallis Test: H [6, *N* = 70] = 41.23, *p* = 0.00001, followed by Multiple Comparison Test: CT21 vs. CT1 [*p* = 0.0004], CT4 [*p* = 0.000003], CT13 [*p* = 0.02], and CT24 [*p* = 0.03], as well as CT19 vs. CT4 [*p* = 0.003] and CT13 [0.04]); *Pdf*^0^: Kruskal–Wallis Test: H [6, *N* = 74] = 20.70, *p* = 0.002, followed by Multiple Comparison Test: CT16 vs. CT1 [*p* = 0.03] and CT19[*p* = 0.03]).

Further, we checked whether the absence of PDF-conveyed information influences the autonomous clock of *Pdf*^0^ photoreceptors (R-cells), which (like the glial cells) belong to the peripheral circadian oscillators. The comparative analysis revealed that daily patterns of changes (in LD) of PER-specific immunofluorescence in the nuclei of *Pdf*^0^ and CS photoreceptor cells were similar (Figure 7A). The average level of immunofluorescence (level of PER), however, was smaller in *Pdf*^0^. Interestingly, this decrease resulted from higher variability in the level of fluorescence in the compound eye photoreceptors (Figure 7B). The standard deviation in *Pdf*^0^ was twice as big as in CS. Therefore, PER molecular oscillations in the retina photoreceptors may be regarded as less synchronized in the absence of PDF. This can be also observed in DD. The level of PER in DD was much lower in *Pdf*^0^ than in CS, showing very small amplitude (Figure 7C). Since we have not observed the R-cells to express PDFRs, they appear to receive this signal indirectly. It may occur through the marginal glia of the proximal lamina, which strongly express PDFRs (Figure 4E).

### Lack of PDF changes the daily pattern of abundance of BRP protein in the lamina synaptic cartridges

**Figure 8:**
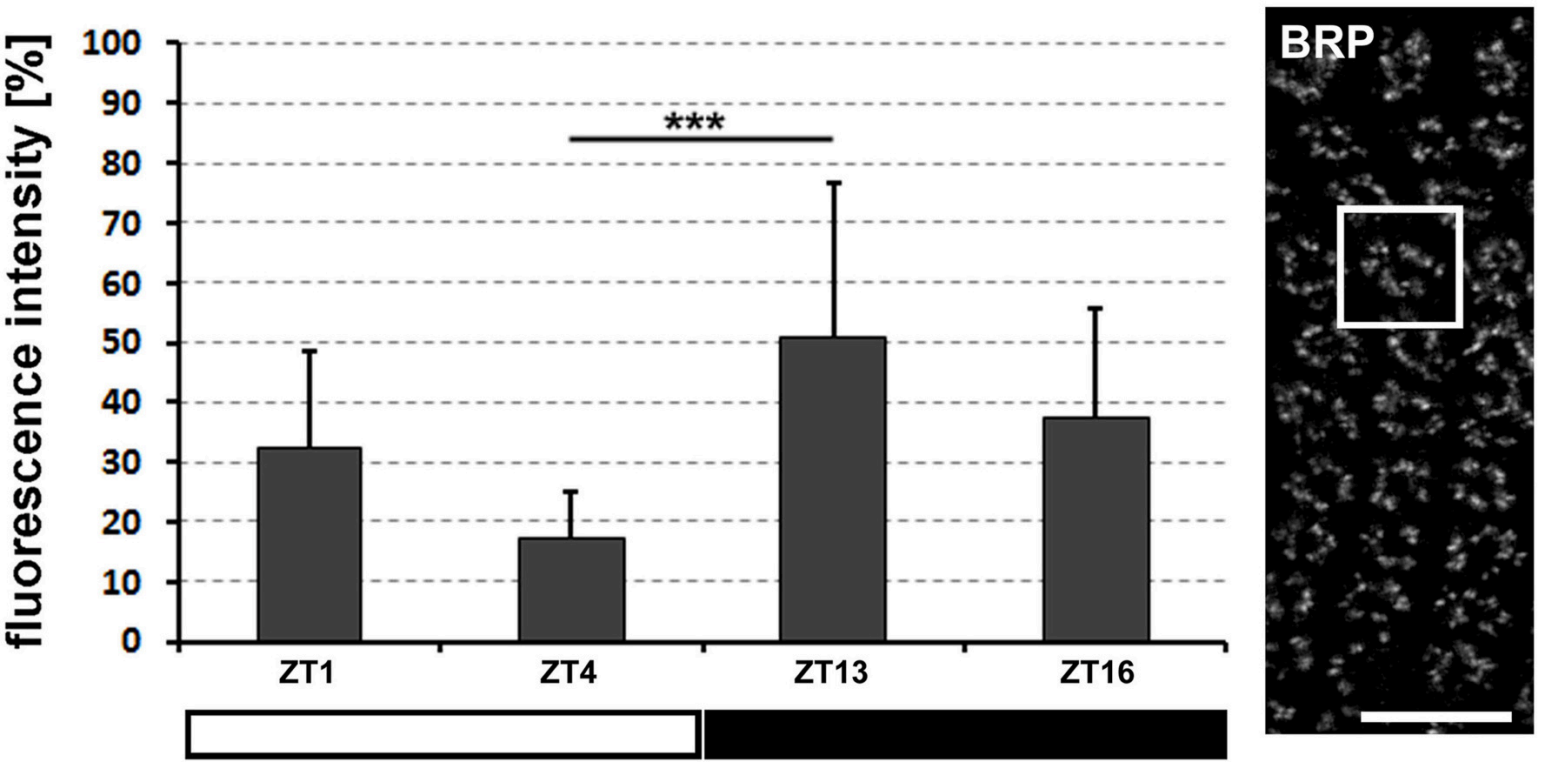
The average level (±*SD*) of BRP-specific immunofluorescence measured in confocal images of the lamina of *Pdf*^0^ mutants at specific time points of LD 12:12. Data represent the average score of fluorescence intensity obtained for the lamina synaptic units called cartridges, which are seen in the lamina fragment on the right (the single cartridge in the frame; ^***^p ≤ 0.001). Scale bar: 10 μm.

Since the lack of PDF in *Pdf*^0^ flies influences PER expression in both the glial and the retinal clocks (although in different ways), we checked the impact of PDF absence on the daily pattern of expression of the presynaptic protein, Bruchpilot (BRP) in the synaptic units (cartridges) of the first visual neuropil (lamina) of *Pdf*^0^ flies. In the lamina neuropil, the BRP daily pattern of expression (composed of two peaks-the morning peak and the evening peak, Górska-Andrzejak et al., 2013), was slightly altered in *Pdf*^0^ flies (Figure 8). While the evening, clock-regulated (glia dependent) peak was firm (there was a statistically significant difference between ZT13 and ZT4; Kruskal–Wallis Test: H [3, *N* = 44] = 15.5, *p* = 0.001, followed by Multiple Comparison Test, *p* = 0.0007), the morning, light-regulated (photoreceptor-dependent) one was small and statistically insignificant (ZT1 vs. ZT4: *p* = 0.2; ZT1 vs. ZT13; *p* = 0.09; ZT1 vs. ZT16: *p* = 0.9). This seems to be consistent with the molecular rhythm strengthening in glia and with de-synchrony that can be observed among the retina photoreceptors.

## Discussion

### Glial oscillators are weaker than neuronal oscillators

The abundance of PER, the prime repressor in the mechanism of circadian clock (Landskron et al., 2009; Hardin, 2011), is known to influence the pace of the clock and the phase of the circadian rhythm (Baylies et al., 1987). Therefore, flies with the lowest levels of PER have slow-running biological clocks (Baylies et al., 1987), while temporary increase in PER concentration can alter the phase of the rhythm (Edery et al., 1994). Our studies revealed lower intensity of PER staining in the optic lobe glial cells than in the l-LNv central pacemakers, as well as in the R-cells of the compound eye, which indicates that the optic lobe glia maintain lower amplitude of PER molecular oscillations. Consequently, they are weaker oscillators with respect to the neurons mentioned above and so they are not able to drive outputs in DD conditions for a longer time (Weiss et al., 2014).

### Glial oscillators are heterogeneous—they express PER at different levels

Glial cells of *Drosophila* are known, however, to be highly diversified both morphologically and functionally (Edwards and Meinertzhagen, 2010). Our studies showed that the large population of glial cells that function as glial clocks (PER-expressing glia) appear to be heterogeneous, as the cells in different locations express different amounts of PER. Because certain oscillatory subgroups (residing in different regions of the optic lobe) display different amplitudes of their molecular rhythms, the strength of their regulatory influence must also differ. Consequently, the lower level of PER in the lamina glia and the higher level of PER in the medulla glia (which is by far the most easily noticeable difference) suggest that the circadian network of these two neuropils require different amounts of the glial circadian activity. Indeed, the epithelial glial cells of the lamina, which envelope the synaptic units (cartridges) of the lamina neuropil (Boschek, 1971; Pyza and Górska-Andrzejak, 2008), might not need to express *per* at high level, as the presynaptic terminals of photoreceptors—the main components of the cartridge—are equipped with their own, autonomous circadian mechanism (Siwicki et al., 1988; Zerr et al., 1990; Cheng and Hardin, 1998).

### dMnGl display the highest level of PER

The highest level of PER in dMnGl, on the other hand, implies that these cells belong to the strongest of glial oscillators. It may also support the hypothesis that PER in glia is required for the regulation of circadian light sensitivity (Zwarts et al., 2015), in which dMnGl could cooperate with their close neighbors, the l-LNv pacemakers (Yoshii et al., 2016). This agrees with the fact that the level of PER in glia, and especially in dMnGl, is elevated in *Pdf*^0^ mutants in LD at ZT1 and in DD at CT1. It is tempting to speculate that in the absence of PDF signaling from the l-LNvs the glial cells increase their PER expression (the strength of their molecular rhythms) to compensate for that lack. In DD conditions, they appear to do so at two crucial time points, at the beginning of the circadian day (regulated predominantly by light) and at the beginning of the subjective night (clock-regulated time point).

### Lack of PDF changes the level of PER in glia

As already mentioned, the main function of the LNv pacemakers is coupling different pacemakers/clock centers within the fly brain (Helfrich-Förster, 1998; Renn et al., 1999). PDF synchronizes the different clock neurons that make up the *Drosophila* circadian neural circuit via PDF receptors (Renn et al., 1999; Lin et al., 2004; Lear et al., 2005; Nitabach et al., 2006; Shafer et al., 2008; Im et al., 2011). It therefore appears as a good candidate for linking up the glial oscillators to the neuronal oscillatory network. This notion agrees with the fact that glial cells take part in the output regulation, since modifications of gliotransmission, calcium stores, or glial ionic gradients result in the arrhythmic locomotor activity (Ng et al., 2011).

Assuming that the highest level of PER specifically in dMnGl results from the direct influence of PDF signaling from the LNvs (Helfrich-Förster, 1998), we checked the glial level of PER in null *Pdf*^0^ mutants, which display reduced morning behavior and advanced evening behavior in LD conditions, as well as progressive dampening of locomotor rhythmicity and shortened period in DD (Yoshii et al., 2016). We found out that *Pdf*^0^ mutants displayed higher level of glial PER than CS. This confirmed the notion that dMnGl belong to multiple targets of PDF but are normally (in CS) negatively influenced by PDF. We conclude this because the level of glial PER is elevated in the absence of PDF. PDF regulation of *per* and *tim* rhythms in *Drosophila* optic neuropils has been reported by Damulewicz et al. (2015). PDF has also been reported to act on PDF neurons themselves to regulate their rhythmic strength (in addition to evening activity phase and period length regulation in non-PDF clock neurons; Lear et al., 2009).

These results also show the range of PDF inhibition. In *Pdf*^0^ mutants, all types of glia exhibit higher level of PER, maintaining differences among the glia of different locations (which are observed in CS). We therefore conclude that PDF influences the entire glial circadian system. Since the amplitude of the molecular rhythm is proportional to the level of PER, the increase in the level of PER in glia in the PDF-free tissue must result in strengthening of the glial circadian functions and, consequently, in increased impact of the glial oscillators on the whole circadian network.

The significantly higher level of PER in glia of *Pdf*^0^ mutant also suggests that the glial cells may play a submissive role in the circadian system. Modulating their amplitude, the PDF-positive clock cells actually influence the gear of circadian clock of the glial cells, which seem to be capable of expressing PER at much higher level when allowed by the LNv pacemakers (the lack of PDF appears to be interpreted by the glial cells as green light for amplitude enhancement).

### PDFR in glia

Till now, the presence of PDFRs have been reported in the PDF and non-PDF clock neurons (Lear et al., 2009; Im and Taghert, 2010) and the non-neuronal (glial) cells situated at the base of the compound eye (Im and Taghert, 2010). Our analysis of the pattern of expression of *pdfR*-Gal4 driver using sequences upstream of the *pdfR*-gene (Lear et al., 2009) revealed that dMnGl express receptors for PDF. On this view, the LNvs can be said to communicate not only to other clock neurons but also to the glial clocks, which can be one of the components of the output of the LNv circuit. The fact that the glial clocks belong to the PDFR-responsive targets indicates their importance for the activity of the whole circadian network. It also implies that the glial clocks have to be synchronized in the same way (via PDF) as different clusters of neuronal pacemakers.

The above findings may explain the observation by Lear et al. (2009), who reported that the rescue of *Pdfr* mutant phenotypes using *Pdf-*Gal4 and *npf-*Gal4 drivers (which do not drive expression to glial cells) failed to rescue significantly both the LD and DD phenotypes. This failure may have been caused by the absence of the relevant glia-derived circadian components.

Judging by the intensity of GFP fluorescence, however, the level of PDFR in glia appears to be lower than in other cells, which may be related to the negative influence of PDF on the level of PER in glia. This mechanism would enable modulation, but at the same time it would protect against the complete switching off the glial circadian functions. The low level of PDFR expression may also explain why dMnGl were not visible when PDFR was detected by the antibodies raised against N- (Hyun et al., 2005) or C-terminus (Mertens et al., 2005). Detection might thus be restricted to the cells with relatively high level of PDFR expression.

The results assessing the level of PER-immunofluorescence in flies with silenced expression of PDFR show the tendency to increase PER level at CT13, similar to the one observed in *Pdf*^0^. They are, however, not entirely conclusive, as the increase observed in the experimental flies is not statistically significant with respect to the control flies. It appears that the RNAi-mediated silencing did not suppress the PDFR expression in glia efficiently enough to mimic the complete lack of PDF in *Pdf*^0^ mutants.

### Glial cells of *Pdf*^0^ display higher level of REPO, the glia specific protein

We observed that *Pdf*^0^ mutants display an increased level of REPO, a glia-specific, paired-like homeodomain transcription factor, which inhibits neuronal and activates glial differentiation during development (Xiong et al., 1994; Halter et al., 1995). The lack of PDF-conveyed signals from the LNvs influences the glial functions controlled by REPO. The function of REPO in the adult brain is still unclear. Recently it has been found, however, that REPO controls glutamate receptor clustering and synaptic physiology at *Drosophila* larval neuromuscular junction (Kerr et al., 2014). Importantly, the expression of glial *repo* is also required for the Long-Term Memory (LTM) formation, as its expression increases upon LTM induction (Matsuno et al., 2015). Artificially elevated REPO expression can also rescue mutants with LTM defects and the knockdowns of KLINGON, the cell adhesion molecule required for LTM formation, which localizes to juncture between neurons and glia. Consequently, REPO influences the KLG-mediated communication between neurons and glia (Matsuno et al., 2015). Higher levels of REPO in dMnGl may, therefore, reflect the higher level of communication. The coincidence of the elevated levels of PER and REPO in *Pdf*^0^ mutants suggests that the circadian clock modulates both REPO-controlled glial functions and neuron-glia interactions.

### R-cells respond to lack of PDF in a different way than glial cells

Photoreceptors of the compound eye (and ocelli) of the fruit fly were among the first, in which robust circadian oscillations of PER and TIM were observed (Siwicki et al., 1988; Zerr et al., 1990). Later studies confirmed that circadian oscillations in the R-cells occur autonomously, i.e., independently of the central circadian pacemaker in the brain (Cheng and Hardin, 1998), being involved in regulation of the visual system sensitivity to daily changes of light intensity (Giebułtowicz, 2000). Our results suggest that the PDF-releasing LNvs, which directly perceive light, influence the circadian oscillations of the retina photoreceptors via PDF signaling. PDF has been known to provide feedback facilitating synchronization of different groups of the clock neurons within the brain (Lin et al., 2004). The primary role of PDF may lie in enhancing and synchronizing individual clock oscillations (Hyun et al., 2005; Mertens et al., 2005). Since different R-cells of *Pdf*^0^ retina displayed major changes in the level of PER nuclear accumulation at the beginning of the day, revealing signs of desynchronization, our results show that the eye multiple oscillators may also be coupled, at least to some extent, via PDF. The eye photoreceptors may thus depend on PDF synchronization. This also explains the necessity of the clock neurons to have direct light input through CRY. On the other hand, the compound eye CRY appears to have a minor contribution to light entrainment. Flies expressing *cry* in the eyes do not entrain significantly better than *cry* mutants (Emery et al., 2000; Yoshii et al., 2016).

### Lack of PDF influences daily pattern of BRP

Daily remodeling of the lamina synaptic contacts has been observed using immunohistochemistry based on Bruchpilot—specific Nc82 antibody (Górska-Andrzejak et al., 2013). The level of Bruchpilot (BRP), the large scaffold protein that is a major constituent of the presynaptic ribbons (so called T-bars) of synapses, fluctuated during the day and night (Górska-Andrzejak et al., 2013). The morning and evening peaks, observed in LD 12:12, are regulated in different ways. The morning peak depends predominantly on the light and phototransduction pathway in the R-cells of the retina and also on the clock gene *per*. The evening peak, on the other hand, is regulated endogenously, by the input from the pacemaker located in the brain. In addition, the two peaks depend on the clock gene-expressing photoreceptors and on the glial cells of the visual system, respectively. Interestingly, in *Pdf*^0^ mutant, the first peak was smaller and insignificant, while the second was still present. The results concerning the level of PER in the glia and the R-cells of *Pdf*^0^ mutant, appear to explain such pattern of BRP expression in these flies. The first peak, which depends predominantly on the presence of the light stimuli and the R-cells activity may be smaller due to visible desynchronization of the compound eye photoreceptors, whereas the second peak is maintained since it is driven by the glial cells, which (judging by increased level of PER in their nuclei) appear to be more active in this mutant than in the wild type flies. Similar results were obtained by Damulewicz et al. (2013) on the expression of the α-subunit of the sodium-potassium pump in *Pdf*^0^ mutants.

PDF-conveyed information is able to reach the R7 and R8 photoreceptors, as they project to the medulla neuropil, where they terminate in different layers of synaptic connections; R7 in layer M6, R8 in layer M3 (Kremer et al., 2017). It is also possible, however, that paracrine release of PDF can reach the proximal lamina, where the terminals of R1-R6 end. It cannot be a coincidence that the receptors for PDF are expressed both in the marginal glia of the proximal lamina (Figure 4E) and in the glial cells situated at the base of the compound eye (Im and Taghert, 2010). The former cells extend their processes toward the distal part of the lamina so high that they branch among processes of the epithelial glial cells and contact the terminals of R1–R6 (Edwards et al., 2012). Although they do not appear to form such intimate connections with photoreceptor terminals as the epithelial glial cells (which invaginate the terminals forming the so called capitate projections, Stark and Carlson, 1986), they do invaginate photoreceptor terminals at some sites of contacts and contain coated vesicles (as shown in EM by Edwards et al., 2012). Small, club-headed capitate projections of glial cells invaginating the terminals of R7 and R8 in the medulla (Takemura et al., 2008; Edwards and Meinertzhagen, 2009) are most probably the projections of dMnGl (Edwards et al., 2012), which we have shown to poses PDF receptors.

To sum up, our studies show for the first time that glial cells in general and dMnGl in particular, belong to PDF downstream circuits as the integral part of the LNv pacemakers output. It is the first step toward understanding how the activity of peripheral oscillators, such as the glial cells or the eye photoreceptor cells, is synchronized with the circadian network and adjusted by the central clock. Further studies should elucidate in greater detail whether other types of glia possess PDF receptors, and dissect the molecular mechanism by which PDF acts on glial cells in order to regulate their clock and circadian activity.

## Author contributions

JG-A: Designed the study, performed experiments, collected and analyzed data, and prepared the manuscript; EC: Performed experiments and collected data; LW: Performed some of the experiments and revised the manuscript; KW: Performed one of the experiments.

### Conflict of interest statement

The authors declare that the research was conducted in the absence of any commercial or financial relationships that could be construed as a potential conflict of interest.
